# Depression in Adolescence and Brain-Derived Neurotrophic Factor

**DOI:** 10.3389/fnmol.2022.947192

**Published:** 2022-07-07

**Authors:** Boyoung Lee, Eunjin Shin, Inwoong Song, Bomi Chang

**Affiliations:** Center for Cognition and Sociality, Institute for Basic Science (IBS), Daejeon, South Korea

**Keywords:** depression, adolescence, BDNF, environmental risk factors, genetic risk factors

## Abstract

The incidence of depression among adolescents has been rapidly increasing in recent years. Environmental and genetic factors have been identified as important risk factors for adolescent depression. However, the mechanisms underlying the development of adolescent depression that are triggered by these risk factors are not well understood. Clinical and preclinical studies have focused more on adult depression, and differences in depressive symptoms between adolescents and adults make it difficult to adequately diagnose and treat adolescent depression. Brain-derived neurotrophic factor (BDNF) is known to play a critical role in the pathophysiology of many psychiatric disorders, including depression. However, there are still few studies on adolescent depression. Therefore, in this review paper, the causes and treatment of adolescent depression and the function of BDNF are investigated.

## Introduction

Adolescence is a period of significant development between puberty and adulthood during which the knowledge and skills necessary for adulthood are acquired (Dahl et al., 2018). Prominent among the brain regions undergoing developmental changes during adolescence are stressor-sensitive areas such as the frontal lobe; therefore, the brain during this period is sensitive to external stress and stimuli, making it vulnerable to anxiety and various neuropsychiatric diseases (Spear, 2000). Major depressive disorder (MDD) in adolescence is one of the most common psychiatric disorders, with a prevalence of 4–5% in mid-late adolescence (Costello et al., 2005; Goldstone et al., 2020). In addition, depression in adolescence is a risk factor for suicide and substance abuse, has a high risk of relapse and often persists as adult depression, placing a great burden on society (Patel et al., 2007). However, the cause of and an effective treatment for depression in adolescence have not been sufficiently elucidated, and depression in adolescence differs from depression in adults in many ways, making it difficult to diagnose and treat MDD in adolescence in a timely manner (Kessler et al., 2001).

There are differences in the symptoms of depression between adolescents and adults, and MDD in adolescence is often left untreated (Rice et al., 2019). For example, adolescents with depression maintain relationships with close friends, while adults are often known to avoid social contact. Additionally, adolescents are more likely to experience frequent drowsiness, and adults are more likely to experience insomnia when depressed (Rice et al., 2019). Depressed adolescents also express their moods and emotions through anger and irritability, unlike depressed adults, who are usually sadder and more withdrawn (Stringaris et al., 2013; Rice et al., 2019). Therefore, biological and objective verification is required for the accurate diagnosis of adolescent MDD. To date, clinical and preclinical studies have focused more on adult depression, and few studies on the mechanisms and treatment of depression in adolescence have been reported. Treatment for MDD in adolescence includes psychotherapy and antidepressants, particularly selective serotonin reuptake inhibitors (SSRIs) (Ryan, 2005). Recently, it has been reported that ketamine, a rapid antidepressant, is effective in the treatment of adolescent depression (Di Vincenzo et al., 2021). However, the mechanisms for the effectiveness of these treatments are still not well understood.

Brain-derived neurotrophic factor (BDNF) is an activity-dependent secreted growth factor that plays an important role in brain development and synaptic plasticity (Park and Poo, 2013; Riffault et al., 2018). Once secreted, the BDNF precursor (proBDNF) is converted to the mature form (mBDNF or BDNF) by proteolytic cleavage (Riffault et al., 2018). A signaling cascade triggered by BDNF and its receptor, the receptor tyrosine kinase TrkB, is known to regulate the development and survival of the central nervous system (Riffault et al., 2018). Indeed, BDNF-deficient mice show developmental defects in the brain and die shortly after birth (Ernfors et al., 1994). BDNF is also important for synaptic plasticity and long-term memory and is known to play a key role in the pathophysiology of many psychiatric disorders, including depression, posttraumatic stress disorder, schizophrenia, and obsessive-compulsive disorder (Boulle et al., 2012). However, studies on the role of BDNF in adolescent depression are limited. Therefore, this review focuses on clinical and preclinical studies on the BDNF signaling pathway as a target for the diagnosis and treatment of adolescent MDD.

## Risk Factors for Adolescent Major Depressive Disorder

Depression is a leading cause of disability worldwide and is projected to become the largest disease burden worldwide by 2030 (World Health Organization, 2011). Despite efforts aimed at improving interventions, the prevalence of depression in adolescence is still increasing. Evidence shows that depression in adolescence is associated with a variety of negative outcomes, including suicidality, social impairment, and poor physical and mental health (Collishaw et al., 2016). However, the causes of this increase in adolescent depression are not yet clear.

Environmental stress in childhood is one of the most significant risk factors for adolescent-onset depression episodes (Hankin, 2015). In the case of environmental factors, stress, abuse, physical or emotional trauma, diabetes, obesity, and other chronic conditions, such as developmental or learning disabilities and traumatic brain injury (TBI), are known factors associated with the development of MDD in adolescence (Lewinsohn et al., 1998). Neonatal stress, childhood stress, and adolescent stress are all important periods in the development of depression in adolescence (Andersen and Teicher, 2008). Research has shown that parental stress, particularly maternal stress during pregnancy, is correlated with the development of depression in children, implying that prenatal stress also affects the development of depression in adolescence (Bergman et al., 2007). Aside from stress, a study found that various factors, such as diet, immunity, and gut bacteria, are involved in the onset of depression (Sandhu et al., 2017).

Many children are exposed to similar levels of stress, but not all children develop depression (Graham-Bermann et al., 2009). Thus, many researchers focus on genetic factors as risk factors for adolescent depression. Regarding genetic factors, several studies have examined a family history of depression. Williamson et al. (1995) investigated whether family members or relatives of depressed adolescents had higher rates of depression than those of healthy adolescents. The study examined 228 first-degree and 736 second-degree relatives of 76 adolescents with MDD and compared them with 107 first-degree and 323 second-degree relatives of 34 healthy control adolescents for the incidence of MDD. The results clearly showed that the relatives of depressed adolescents had significantly higher lifetime rates of MDD than the first-degree relatives of healthy controls. More interestingly, the first-degree relatives of depressed adolescents who were also suicidal had increased lifetime rates of suicidal behavior, which significantly co-segregated with MDD, providing evidence for the familial aggregation of depression in adolescent-onset MDD. Twin studies also confirmed the association of genetic factors in adolescent depression, although there are very few longitudinal twin studies of adolescent depression (Rice, 2009; Xia and Yao, 2015). Although this study was conducted in adolescent females, the only twin study reported to date confirmed that the prevalence of MDD in adolescent co-twins was much higher (up to 36%) in monozygotic twins than in dizygotic twins. In addition, the onset of MDD in co-twins was reported to be much earlier in monozygotic twins (Glowinski et al., 2003). In addition, clinical and preclinical studies have disclosed the association of depressive symptoms in fathers or mothers with depressive symptoms in their adolescent offspring, suggesting genetic transmission from parent to offspring (Chubar et al., 2020). Taken together, these findings show that genetic factors are important risk factors for adolescent depression.

There is interesting research that identified the longitudinal nature of depression symptom trajectories in adolescence and their associations with genetic and environmental risk factors in early childhood and adolescence. Five depression symptom trajectories were derived from 3,525 individuals (Savage et al., 2015). First, the stable-low trajectory involved individuals who had consistently low levels of depression symptoms. Second, the early-adult–onset trajectory involved individuals who started with low depression symptoms that increased during adolescence and young adulthood. Third, the adolescent-limited trajectory involved individuals who experienced elevated levels of depression symptoms only during adolescence, and fourth, the childhood-limited trajectory involved individuals who started with elevated levels of depression symptoms in childhood that decreased. Fifth, the childhood-persistent trajectory involved individuals with moderate levels of depression symptoms that continued to increase and stayed high during adolescence and into young adulthood. Interestingly, the authors described “less favorable trajectories” for the early-adult–onset trajectory and the childhood-persistent trajectory because depression symptoms persist into adulthood. Importantly, the study confirmed that these two less favorable trajectories were significantly associated with a combination of genetic and environmental risk factors. Instead, it was confirmed that the time-limited trajectories with the disappearance of depressive symptoms in adults were not associated with a genetic factor. Therefore, looking at the combination of genetic and environmental risk factors will help identify groups with chronic and severe depressive symptoms for more aggressive treatments. Taken together, these findings show that the modulation of genetic factors during adolescent depression may be important in controlling the persistence of depressive symptoms.

## Brain-Derived Neurotrophic Factor in the Development of Adolescent Major Depressive Disorder

Cumulative evidence from studies to identify biomarkers associated with depressive disorder suggests that BDNF is highly associated with depressive disorder and has potential in diagnostic and prognostic evaluation and screening for high-risk groups among those with adolescent depressive disorder (Schmidt et al., 2011; Bilgiç et al., 2020; Lee et al., 2021). In adults, decreased BDNF expression at the mRNA and protein levels in both blood samples (Sen et al., 2008) and postmortem brain tissues (Pandey et al., 2008; Mariani et al., 2021) from depressed individuals has been reported. Consistent with the clinical findings for BDNF expression in depression, in rodents, different stress paradigms that can induce depressive-like behaviors, such as chronic stress, social defeat, or exposure to pre- and perinatal stress, also affect BDNF mRNA and protein levels in various brain regions, including the hippocampus and the prefrontal cortex (Krishnan et al., 2007; Yu and Chen, 2011; Stepanichev et al., 2014; Zaletel et al., 2017; Wei et al., 2018). In addition, treatment with antidepressants such as SSRIs and the rapid-acting antidepressant ketamine can upregulate BDNF expression and normalize reduced BDNF blood levels in human MDD patients and animal models (Duman et al., 2016; Zhou et al., 2017).

**FIGURE 1:**
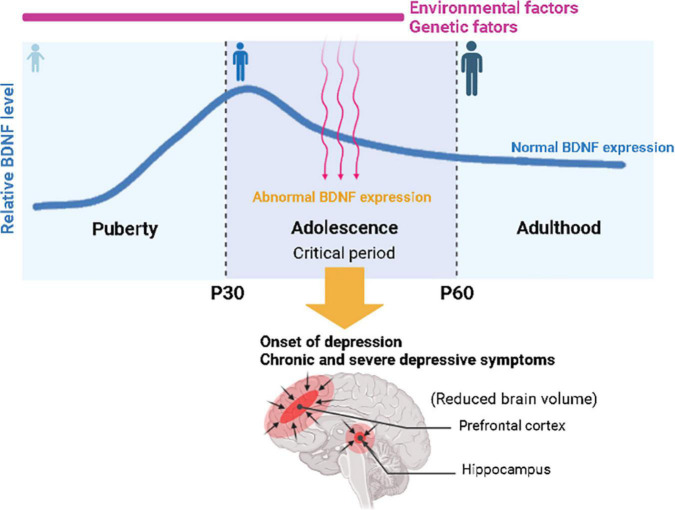
Schematic illustration of the association between environmental and genetic risk factors related to brain-derived neurotrophic factor (BDNF) expression for the onset of depression in adolescence. Relative BDNF levels peak in early adolescence and gradually decrease with age. Abnormal BDNF expression due to genetic and environmental factors during adolescence may trigger the onset of depression and lead to chronic and severe depressive symptoms, with a risk of a smaller prefrontal cortex and hippocampus, leading to the susceptibility to mood disorders.

Similar to adults, the association between BDNF and adolescent MDD was confirmed in humans (Zhao et al., 2018; Lee et al., 2020) and animal models (Chen et al., 2012). To investigate whether the BDNF gene is associated with the development of MDD in young patients, several groups have performed polymorphism analyses using targeted DNA sequencing and identified a human single-nucleotide polymorphism (SNP), BDNF Val66Met, that was associated with the development of adolescent MDD (Lau et al., 2010; Cruz-Fuentes et al., 2014). The BDNF Val66Met polymorphism (rs6265) inhibits the activity-dependent release of BDNF and has been linked to reduced hippocampal volume and increased susceptibility to anxiety and depressive behaviors (Molendijk et al., 2012). Interestingly, Dincheva et al. (2017) demonstrated that BDNF Met/Met mice showed normal behavior at postnatal day 30 (P30), which is an early adolescent period, but anxiety behavior was increased in young adults (at P60), indicating that disrupted BDNF signaling due to the BDNF polymorphism mostly affected behaviors related to mood disorders during the adolescent period. Given previous findings that BDNF expression peaks in the hippocampus and prefrontal cortex during adolescence and gradually decreases with age, these results suggest that decreased BDNF expression and function during adolescence may be a major risk factor for the development of depression and other mood disorders, such as anxiety disorder (Figure 1; Dincheva et al., 2016). These studies suggest that individuals with the BDNF Val66Met polymorphism might be at risk for a smaller prefrontal cortex and hippocampus, leading to the susceptibility to mood disorders, which strengthens the hypothesis that BDNF plays an important role in depression (Montag et al., 2009; Yu and Chen, 2011; Duman, 2017). In addition, rumination has also been reported as a factor associated with cognitive vulnerability in depression in adolescents (Hankin, 2008; Jacobs et al., 2008). Genetic studies examining candidate genes for rumination in children and adolescents confirmed that variations in the BDNF and serotonin genes were associated with rumination propensity, suggesting a possible role for BDNF as a biomarker for the incidence of rumination regarding life stress as well (Beevers et al., 2009; Zwolinska et al., 2021). Moreover, the effect of stress on BDNF expression in adolescence was found to be different from that in adulthood in rodents (Bath et al., 2013; Lee et al., 2020). For example, in the hippocampus of mice, BDNF expression increased after social defeat in adolescence but not in adulthood (Coppens et al., 2011; Lee et al., 2020). Maternal separation stress after early birth transiently increased cortical BDNF expression during adolescence, and then BDNF levels decreased in adulthood (Lee et al., 2020). Therefore, differences in BDNF expression between adolescence and adulthood may be related to differences in stress-driven symptoms between adolescents and adults (Lee et al., 2020).

How might BDNF dysfunction play a more important role in the pathophysiology of depression in adolescents than in adults? Serotonin [5-hydroxytryptamine (5-HT)] dysfunction is closely related to BDNF expression and release in adolescent depression (Szapacs et al., 2004). Studies have shown that 5-HT fiber density in the prefrontal cortex was decreased between P21 and P42 in BDNF Met/Met mice compared to wild-type mice (Szapacs et al., 2004; Duman, 2017). Surprisingly, the administration of fluoxetine during critical P21–P42 adolescence but not during the P60–P81 period normalized reduced 5-HT fiber density in the prefrontal cortex of adult BDNF Met/Met mice, indicating that altered BDNF-expression-mediated 5-HT dysfunction during early adolescence may be important in the pathophysiology of mood disorders, including depression and anxiety disorders, during adolescence (Szapacs et al., 2004; Duman, 2017). In addition, the morphological and biochemical properties of γ-aminobutyric acid (GABA) inhibitory synapses display profound changes and obtain mature properties in some areas of the brain during adolescence (Caballero and Tseng, 2016). Importantly, a study reported that BDNF Met/Met mice exhibited a robust reduction in somatostatin (SST) cell density in the dorsal hippocampus, and adolescent CORT treatment in BDNF Met/Met mice further significantly reduced SST and parvalbumin (PV) dendrite spine density, indicating that BDNF is important not only for the normal development of GABAergic neurons but also for the pathological condition of GABAergic neurons induced by stress in adolescence (Hill et al., 2020).

Although reduced BDNF expression and function are highly associated with depression in animal models and human patients, there are inconsistent results regarding the association between BDNF levels and depression during adolescence. Some studies identified increased serum BDNF levels in adolescents with MDD compared with healthy controls (Coppens et al., 2011; Bilgiç et al., 2020). Thus, a decrease or increase in BDNF levels appears to be associated with depression during adolescence. Further studies are needed to understand whether increased BDNF levels in patients with MDD are directly related to MDD progression in adolescence. Overexpression of BDNF during the adolescence period in animal models could be a useful approach to address this question.

## Brain-Derived Neurotrophic Factor in the Treatment of Adolescent Major Depressive Disorder

Antidepressants play a key role in the treatment of MDD owing to their verified efficacy and wide availability (Block and Nemeroff, 2014; Luo et al., 2020). Throughout a 20-year period, SSRIs were stably prescribed for approximately 70% of patients with MDD and among SSRIs, sertraline and fluoxetine were the most prescribed antidepressants. For adolescent MDD, SSRIs are also the major prescribed antidepressants, and the efficacy and underlying mechanisms of their effectiveness in adolescent MDD are being carefully examined (Luo et al., 2020). Ketamine is a non-competitive antagonist of the N-methyl-D-aspartate glutamate receptor and has traditionally been used as a surgical anesthetic (Krystal et al., 2019). In the early 2000s, the first clinical study reported that a single intravenous infusion of subanesthetic doses of ketamine had potent, rapid and sustained antidepressant effects in individuals with treatment-resistant depression (Krystal et al., 2019). In particular, because of its very rapid response, ketamine has since been extensively tested as a rapid antidepressant, and recently esketamine, an isomer of ketamine, has been approved by the U.S. Food and Drug Administration as a nasal spray for patients with treatment-resistant depression (Krystal et al., 2020; Kaur et al., 2021). Therefore, the efficacy of ketamine for adolescent MDD in preclinical and clinical studies is also being investigated (Strong and Kabbaj, 2018; Dwyer et al., 2021). Exercise and other types of behavioral therapy are also applied to young patients with depression (Hughes et al., 2013; Oberste et al., 2018).

Several studies have suggested that normal BDNF signaling is required for antidepressant action (Castrén and Monteggia, 2021). Antidepressants induce plastic changes in the rodent brain that may be associated with changes in BDNF levels and/or BDNF function (Kozisek et al., 2008; Castrén and Monteggia, 2021). However, in adolescents, changes in BDNF levels during antidepressant treatment are relatively unknown. Lee et al. (2020) reported that rats treated with escitalopram, an SSRI, twice a day for 4 days showed significantly increased BDNF mRNA and protein levels and TrkB mRNA levels compared to control rats, but rats treated with desipramine, a tricyclic antidepressant (TCA), did not show increased BDNF or TrkB. The failure of desipramine to increase BDNF and TrkB levels in juvenile rats is consistent with the lack of efficacy of desipramine in children and adolescents, suggesting a requirement for BDNF activation for antidepressants to be effective in the treatment of adolescent depression (Kozisek et al., 2008; Lee et al., 2020). In addition, as we discussed earlier, fluoxetine was effective when it was administered during early adolescence but not during the adult period in BDNF Met/Met mice, demonstrating that it is essential to optimize the timing of pharmacological interventions to determine the potential beneficial effects of SSRI treatment during adolescent periods (Dincheva et al., 2017).

Brain-derived neurotrophic factor levels and their early changes during adolescence may also predict antidepressant response in adults with MDD. Lee et al. (2020) investigated whether changes in BDNF levels in serum can predict responsiveness to antidepressants, particularly fluoxetine, in adolescents with MDD. The study identified that a decrease in serum BDNF levels in the early phase of SSRI treatment is associated with a later SSRI response in adolescents with MDD, suggesting that BDNF can be a critical biomarker to predict the effectiveness of antidepressants (Lee et al., 2020). More studies are needed to identify an association between the transient decrease in serum BDNF levels during adolescence and later antidepressant effectiveness. It is also important to note that BDNF exerts antidepressant-like effects in the hippocampus and prefrontal cortex but may have different or even opposite effects in other brain regions, such as the amygdala and nucleus accumbens (NAc), in which chronic stress increases BDNF expression (Yu and Chen, 2011). BDNF infusion into the NAc exerts a depressive effect, and the blockade of BDNF function in the NAc exerts an antidepressant-like effect (Yu and Chen, 2011; Shirayama et al., 2015). Thus, the differential role of BDNF in depression could be attributed to its location in depression-related circuits. Therefore, temporal and spatial regulation of BDNF expression may be important for understanding the mixed results of BDNF and antidepressants in adolescent depression.

In addition to SSRIs, the glutamate system has been implicated in the pathophysiology of adult MDD (Niciu et al., 2014). Recently, ketamine, a glutamate modulator, has attracted great attention due to its rapid antidepressant effect in adults (Frizzo, 2019). Ketamine also appears to be effective in reducing anhedonia and suicidality in adolescent MDD patients, making ketamine a potentially valuable new treatment option for adolescents (Dwyer et al., 2021; Yavi et al., 2022). An increase in BDNF mRNA or protein levels in the hippocampus and prefrontal cortex by ketamine treatment has been reported in animal models of depression (Autry et al., 2011), and results from clinical studies of an increase in serum BDNF with ketamine treatment have also been reported in MDD patients (Strasburger et al., 2017; Arosio et al., 2021). Thus, both SSRIs and ketamine appear to share some common mechanism through the BDNF signaling pathway for antidepressant action (Strasburger et al., 2017; Zanos et al., 2018). However, the time for an effect to appear after SSRI and ketamine treatment varies. In the case of SSRIs, it is known that it takes 2 weeks to 2 months for an effect to appear, but in the case of ketamine, an effect is known to appear as early as 4 h after administration (Zanos et al., 2018). Therefore, it may be important to test temporally specific changes in BDNF gene or protein expression in different brain regions or circuits to elucidate the detailed molecular mechanisms for the antidepressant action of SSRIs and ketamine (Bjorkholm and Monteggia, 2016; Song et al., 2017).

Electroconvulsive shock therapy, exercise or endurance and muscle-strengthening training are also considered treatments for depression in adults, and these treatments are also applicable in the treatment of depression in adolescents (Strohle, 2009; Wachtel et al., 2011; Hughes et al., 2013; Gokce et al., 2019; Wang et al., 2022). These treatments have shown positive effects on adolescent depression, and BDNF has been considered to play a role in their antidepressant actions (Tsai et al., 2010; Murawska-Ciałowicz et al., 2021). For example, depression associated with the BDNF Val/Val variant appears to be more amenable to exercise therapy than depression associated with the BDNF Met/Met variant, which suggests that these non-pharmacological therapies may also have antidepressant effects through a BDNF signaling pathway mechanism (Tsai et al., 2010). Therefore, careful examination of the amount and time of BDNF expression after the pharmacological or non-pharmacological interventions mentioned above will be very helpful in the selection of the appropriate time and treatment method for the treatment of adolescent depression. In addition, understanding the upstream modulators that regulate BDNF expression will help develop tailored treatment strategies.

To understand the upstream modulators regulating BDNF expression in the pathophysiology of depression or after antidepressant treatments, epigenetic and posttranslational modifications to BDNF may be good targets. A twin study investigated differences in DNA methylation between twins with or without a lifetime history of early-onset major depression (MD). The study identified genome-wide DNA methylation biomarkers associated with early-onset MD among monozygotic twins, indicating an association between the early life major depression and epigenetic factors (Roberson-Nay et al., 2020). Recently, BDNF glycosylation has been shown to have some implications in depression (Mizui et al., 2019; Yamagata and Nakagawa, 2020). Interestingly, it is known that the glycosylation of pro-BDNF, a precursor of BDNF, is involved in BDNF maturation (Barreda Tomas et al., 2020). However, no studies investigating glycosylated pro-BDNF as a biomarker of depression have been reported. Although designing a basic study of glycosylation is difficult, since asparagine 123 in pro-BDNF is the only N-linked glycosylation consensus site in the BDNF protein (Lessmann and Brigadski, 2009), it may be possible to test whether N-glycosylation at this specific site is altered in adolescent depression or after treatment in patients or animal models by using liquid chromatography–mass spectrometry (LC/MS) techniques. Analyses of glycosylation and glycosylation-related genes for BDNF site-specific glycosylation can help in the identification of future biomarkers of adolescent depression.

## Conclusion

This review focused on clinical and preclinical studies of the BDNF signaling pathway for the diagnosis and treatment of adolescent MDD. The causes and treatment of adolescent depression have not been fully elucidated, and adolescent depression is different from adult depression in many ways, making it difficult to diagnose and treat it in a timely manner. For an accurate diagnosis and appropriate treatment of adolescent MDD, it is important to identify the underlying mechanism of adolescent depression. Since regulated expression of BDNF is well known in adult patients who take antidepressants, we consider BDNF an important target for the treatment for adolescent MDD. Although there are mixed results regarding the BDNF signaling pathway for the onset of MDD and the treatment of adolescent MDD, we believe that understanding the temporal and spatial regulation of BDNF expression provides a clear understanding of the role of BDNF in the pathophysiology of depression. Furthermore, understanding upstream regulators of BDNF expression, such as epigenetic or post-translational modifications like N-glycosylation on pro-BDNF, will help to suggest future biomarkers and therapeutic targets of adolescent depression.

## Author Contributions

BL, ES, and IS provided the article ideas and drafted the manuscript. BC edited the manuscript and designed the schematic illustration for Figure 1. All authors read, edited, and approved the final manuscript.

## Conflict of Interest

The authors declare that the research was conducted in the absence of any commercial or financial relationships that could be construed as a potential conflict of interest.

## Publisher’s Note

All claims expressed in this article are solely those of the authors and do not necessarily represent those of their affiliated organizations, or those of the publisher, the editors and the reviewers. Any product that may be evaluated in this article, or claim that may be made by its manufacturer, is not guaranteed or endorsed by the publisher.
